# Supraspinatus Tendon Thickness Is Associated With Collagen Disorganization in Symptomatic and Asymptomatic Shoulders of Individuals With Unilateral Rotator Cuff Tendinopathy

**DOI:** 10.1002/jor.70200

**Published:** 2026-04-03

**Authors:** M. Chris Zipser, Oscar Vila‐Dieguez, Sebastian Klich, Greg Bashford, Kira Chow, Lori A. Michener

**Affiliations:** ^1^ Division of Biokinesiology and Physical Therapy, Keck Hospital of USC University of Southern California, Los Angeles Los Angeles California USA; ^2^ Department of Orthopaedic Surgery UC San Diego School of Medicine University of California, San Diego La Jolla California USA; ^3^ Department of Sport Didactics Wroclaw University of Health and Sport Sciences Wroclaw Poland; ^4^ Department of Biological Systems Engineering University of Nebraska Lincoln Nebraska; ^5^ Department of Radiology West Los Angeles Veterans Affairs Medical Center Los Angeles California USA; ^6^ Division of Biokinesiology and Physical Therapy University of Southern California Los Angeles California USA

**Keywords:** peak spatial frequency radius, rotator cuff tendon, supraspinatus, tendinopathy, tendon hypertrophy, ultrasound

## Abstract

Rotator cuff (RC) tendinopathy is a common source of shoulder pain and is frequently linked to structural changes in the supraspinatus tendon of greater thickness and disrupted collagen organization. This study aimed to characterize differences in tendon thickness and collagen organization between individuals with unilateral symptomatic RC tendinopathy, their contralateral asymptomatic shoulder, and asymptomatic controls, and to examine the relationship between thickness (macro‐morphology) and collagen organization (micro‐morphology). This is a cross‐sectional observational study of 64 participants diagnosed with RC tendinopathy compared to 64 asymptomatic controls. On ultrasound imaging of the tendon, thickness was measured, and collagen organization was quantified via spatial frequency analysis of peak spatial frequency radius (PSFR). Compared to controls, individuals with RC tendinopathy showed significantly greater tendon thickness on the involved side (4.3 ± 0.7 vs. 3.9 ± 0.5 mm, *p* = 0.0097), but no differences in PSFR. Participants with RC tendinopathy showed no significant differences between sides in tendon thickness or PSFR. In the RC tendinopathy group, tendon thickness and PSFR were inversely correlated on both the involved (*r* = −0.24, *p* = 0.060) and uninvolved (*r* = −0.32, *p* = 0.01) sides, indicating that increased tendon thickness was associated with decreased collagen organization—an association not observed in controls. Findings suggest tendon thickness reflects maladaptive structure in RC tendinopathy, and collagen organization alone may not sufficiently identify tendon pathology. The relationship between tendon thickness and collagen disorganization in RC tendinopathy indicates that tendon maladaptation differs from adaptive hypertrophy in healthy shoulders.

**Statement of Clinical Significance:** Combined evaluation of tendon hypertrophy and collagen organization can enhance structural characterization in those with tendinopathy.

## 1 Introduction

Shoulder pain is estimated to be the third most common reason patients seek care for musculoskeletal pain in primary care [1]. The median global prevalence of shoulder pain is 16%. Rotator cuff (RC) tendinopathy is the most common reason for musculoskeletal shoulder pain [2, 3], and the supraspinatus is the most commonly involved tendon. In prior studies, patients with RC tendinopathy have predominantly shown greater tendon thickness, while deficits in collagen organization have been less consistently identified [4, 5, 6, 7].

Ultrasound imaging offers a non‐invasive, accessible, and cost‐effective method for evaluating tendon structure in both healthy and injured populations. It enables real‐time assessment of tendon macro‐morphology, which can reflect tendon thickening or inflammation commonly seen in RC tendinopathy [4, 5, 6, 7]. Simultaneously, ultrasound allows for analysis of micro‐morphological features, including collagen fiber organization. Computational techniques like spatial frequency analysis provide objective quantification of collagen organization via analysis of speckle pattern of grayscale on the images [8, 9, 10, 11, 12]. In healthy tendons, collagen fibers are typically well‐aligned and organized, whereas injured tendons often display greater thickness and collagen disorganization defined by heterogeneity in the fiber pattern. Using both macro‐ and micro‐morphology measures can enhance the structural characterization of RC tendinopathy and inform clinical decision‐making.

Micromorphological analysis has gained interest for its potential to quantify tendon disorganization. However, Pozzi et al. found no significant difference in peak spatial frequency radius (PSFR) values between individuals with RC tendinopathy and healthy controls, despite clear differences in symptoms and disability [10]. In contrast, studies in other tendons, such as the Achilles and patellar, have demonstrated that PSFR can detect micromorphological abnormalities [8, 12, 13]. This discrepancy between tendons may be attributed to the unique curvature, anatomical location, and loading patterns of the supraspinatus tendon. In RC tendinopathy, assessing PSFR in tandem with tendon thickness may help explain previously inconclusive PSFR findings [4, 5, 6, 7, 14]. Despite growing interest in tendon imaging, few studies have simultaneously examined both macro‐ and micro‐morphological characteristics in RC tendinopathy. Prior work has often focused on a single structural dimension of tendon thickness, limiting the ability to capture the complex, multifactorial nature of tendon pathology. The current study addresses this gap by leveraging ultrasound to comprehensively evaluate both tendon thickness and collagen organization across the tendon image. Analyzing intra‐individual differences (affected vs. unaffected side), as well as group comparisons (tendinopathy vs. control), provides a robust structural profile of tendon health. Assessing the correlation between macro‐ and micro‐morphology metrics allows for new insights, clarifying if tendon thickening is a positive adaptation or maladaptive, by defining the relationship to collagen organization. Our study objectives were to: (1) compare tendon thickness and collagen organization in patients with unilateral RC tendinopathy vs. 1‐1 matched asymptomatic control shoulders, (2) compare tendon thickness and collagen organization between symptomatic and asymptomatic shoulders in RC tendinopathy patients, and (3) correlate tendon thickness with collagen organization in all groups. We hypothesized that participants with RC tendinopathy would demonstrate higher tendon thickness and lower PSFR values on their symptomatic shoulder compared to healthy controls and their asymptomatic shoulder, and the two metrics of tendon structure would be correlated in those with RC tendinopathy.

## 2 Methods

This cross‐sectional observational study involved participants with RC tendinopathy and bilateral asymptomatic controls. We recruited participants diagnosed with RC tendinopathy from local physical therapy and physicians' offices, community centers, and fitness facilities using study flyers, social media advertisements, the clinical data warehouse, online message boards within the institution web domain, and internal email lists. Potential participants expressing interest via email or phone received information about eligibility requirements and procedures, and completed the first‐level screening via questionnaires. After confirming their first‐level eligibility, they were scheduled for an in‐person screening visit and reviewed and signed an electronic informed consent form. This study was approved by the Institutional Review Board, University of Southern California #HS‐20‐00994 (Tables 1 and 2).

**TABLE 1:** Study participant demographics.

Variable	Controls *N* = 64	RC tendinopathy *N* = 64	*p*‐value
Weight	150.8 ± 28.1	152.1 ± 28.0	0.796
Height (in.)	67.3 ± 3.7	66.9 ± 3.7	0.547
BMI	23.3 ± 3.0	23.8 ± 3.6	0.355
Age	28.4 ± 7.3	28.9 ± 8.1	0.723
Dominant shoulder painful	N/A	42	N/A
Weeks since onset		Median: 52 weeks Interquartile range (IQR): 21–130 weeks Range: 4–520 weeks Mean ± SD: 97.9 ± 120.6 weeks	
Penn pain	29.8 ± 0.5	22.3 ± 4.0	< 0.01
Penn satisfaction	9.4 ± 0.8	4.3 ± 2.1	< 0.01
Penn function	59.5 ± 1.0	46.5 ± 7.0	< 0.01
Penn total	98.8 ± 1.8	73.1 ± 10.2	< 0.01

Variable	Controls	RC tendinopathy	*p*‐value
Weight	150.8 ± 28.1	152.1 ± 28.0	0.796
Height (in.)	67.3 ± 3.7	66.9 ± 3.7	0.547
BMI	23.3 ± 3.0	23.8 ± 3.6	0.355
Age	28.4 ± 7.3	28.9 ± 8.1	0.723
Dominant shoulder painful	N/A	42	N/A
Weeks since onset		Median: 52 weeks Interquartile range (IQR): 21–130 weeks Range: 4–520 weeks Mean ± SD: 97.9 ± 120.6 weeks	
Penn pain	29.8 ± 0.5	22.3 ± 4.0	< 0.01
Penn satisfaction	9.4 ± 0.8	4.3 ± 2.1	< 0.01
Penn function	59.5 ± 1.0	46.5 ± 7.0	< 0.01
Penn total	98.8 ± 1.8	73.1 ± 10.2	< 0.01

**TABLE 2:** Tendon thickness (TT) and peak spatial frequency radius (PSFR).

Variable	Controls	RC tendinopathy	*p*‐value	Mean difference (95% CI)
TT (involved)	3.9 ± 0.5	4.3 ± 0.7	0.0097	0.4 (0.07–0.51)
TT (uninvolved)	3.97 ± 0.6	4.1 ± 0.8	0.3310	0.13 (−0.12–0.37)
PSFR (involved)	1.6 ± 0.1	1.6 ± 0.2	0.6367	0.0 (−0.06–0.04)
PSFR (uninvolved)	1.6 ± 0.1	1.6 ± 0.1	0.3339	0.0 (−0.07–0.02)

Participants recruited for this study were 64 with RC tendinopathy and 64 age‐, sex‐, dominance‐, and BMI‐matched controls. Inclusion criteria for both groups were: age 18–55 years old (> 55 were excluded due to increased likelihood of a full‐thickness tendon tear [15]). Inclusion criteria for participants with RC tendinopathy included: (1) Penn Shoulder Score ≤ 85/100 (100 = no disability), (2) unilateral diagnosis of RC tendinopathy via three of five positive clinical tests (Hawkins, Neer, painful arc, empty can, and painful external rotation resistance) [16, 17, 18]. Exclusion criteria for both groups included: (1) presence of neck/thoracic pain or shoulder pain reproduced with Spurling's, cervical rotation, or axial compression tests [19], (2) prior surgery on the shoulder, neck, or thoracic spine, (3) primary adhesive capsulitis: passive range of motion loss > 50% in external rotation or elevation [17, 20], (4) shoulder instability indicated by a positive anterior or posterior apprehension test [17, 20], and (5) full‐thickness RC tear, verified by ultrasound imaging [15].

### 2.1 Procedures

After consent was obtained, a physical therapist performed the clinical tests for RC tendinopathy, and ultrasound imaging to exclude full‐thickness RC tears. A musculoskeletal radiologist reviewed the ultrasound cineloops to confirm if a full‐thickness RC tear was present. Patients completed surveys for demographics and patient‐reported disability.

### 2.2 Patient‐Reported Disability

*The Pennsylvania Shoulder Score (PENN)* consists of three sections: pain, function, and satisfaction. The Penn has demonstrated reliability, validity, and responsiveness in patients with shoulder pain [21, 22]. The average MDC is 12.1‐points and MCID is 11.4‐points [21].

### 2.3 Tendon Morphology

**FIGURE 1:**
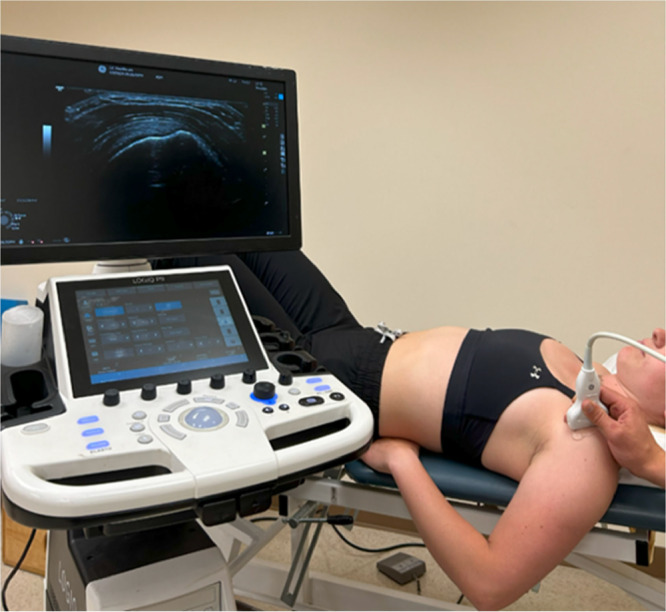
Long axis image acquisition of the supraspinatus tendon for thickness measurement.

Ultrasound images (0.0043 mm/pixel) of the supraspinatus tendon were obtained with a 6–12 MHz linear transducer (P9; GE Healthcare, Wauwatosa, WI). Gray‐scale B‐mode images were obtained with the participant in supine position, and with the arm over the edge of the table in the crass position to expose the tendon (Figure 1). The transducer was placed on the anterior aspect of the shoulder, parallel to the tendon. Two cine‐loops were captured while optimizing the transducer to ensure maximal tendon visualization by ensuring the tendon borders and attachment to the humerus. Specifically, tendon borders were defined inferiorly as the first hyperechoic region above the anechoic articular cartilage of the humeral head, and the hyperechoic superior border of the tendon before the anechoic subdeltoid bursa, and attachment on the humeral tuberosity. Four images were later extracted from these two cine‐loops for analysis.

### 2.4 Macro‐Morphology

**FIGURE 2:**
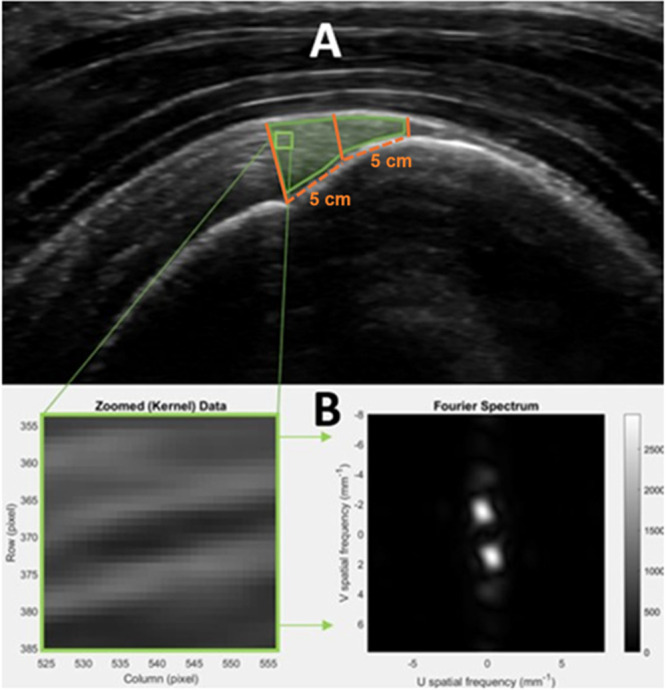
Data analysis procedure for extraction of SFA parameters from images. (A) ROI drawing and kernel example (thinner green) and tendon thickness measurement locations (orange). Solid lines indicate the three measurements that were taken: First line to the left indicates measurement at the anatomical neck of the humerus. Middle solid line at 5 cm from the anatomical neck, and solid line to the right 10 cm from the anatomical neck. Dashed orange line represents distance between measurement landmarks. (B) Zoomed kernel and corresponding 2D Fourier spectrum. The U spatial frequency axis represents lateral (horizontal) variation in the kernel, while the V spatial frequency axis represents axial (vertical) variation along the ultrasound beam, The dominant peak in the Fourier spectrum indicates the primary spatial frequency of the speckle pattern, and its radial distance from the origin corresponds to the peak spatial frequency radius (PSFR), a measure of collagen organization.

Tendon thickness was measured at 5, 10, and 15 mm proximal to the tendon attachment to the greater tuberosity in the longitudinal view. First, a line was drawn from tendon insertion on the humeral head to the articular cartilage junction. Next, from this first line, 3 perpendicular lines were at 5, 10, and 15 mm from the tendon insertion (Figure 2). The three measurements were averaged to represent tendon thickness [5, 23]. Test–retest reliability (ICC = 0.97; minimal detectable change (MDC) = 0.3 mm) was similar to prior studies [5, 24, 25].

### 2.5 Micro‐Morphology

A polygonal region of interest (ROI) within the longitudinal image was selected first (Figure 2). The ROI for the spatial frequency analysis (SFA) was drawn from the anatomical neck of the humerus superiorly to the border of the bursa, following it distally to the apex of the greater tuberosity, and following the lower border of the tendon medially back to the anatomical neck (Figure 2A). All images were processed using previously described custom MATLAB algorithms (Mathworks, Natick, MA) and extraction of SFA parameters [26]. Briefly, the spatial spectrum for each kernel was estimated using a 2D Fast Fourier Transform (FFT) applied to the image kernel. Each kernel was configured as a square of 32 × 32 pixels (1.4 × 1.4 mm), consistent across all window depths, and allowed to overlap in axial and lateral directions within the primary ROI (Figure 2B). Before applying the FFT, kernels were weighted with a spatial Hanning window and zero‐padded in both dimensions to 128 × 128 pixels to increase frequency sampling. After the FFT, a 2D high‐pass filter with a −3 dB cut‐off of 1.0 mm^−1^ was applied to reduce low spatial frequency artifacts. Spatial frequency parameters were then extracted for all kernels within the ROI and averaged for the final SFA statistics. Parameter extraction was repeated for four images per participant, and the average was used as the final value. PSFR was used as the micromorphology parameter. The PSFR is the distance between the origin and the spatial frequency peak in the 2D spatial spectrum, and it represents the dominant spacing of the speckle pattern generated by tissue fascicles/fibers. Higher PSFR values indicate more well‐aligned organization of the collagen fibers [27, 28]. Test–retest reliability was established for this measure via our own internal testing (ICC = 0.75; MDC = 0.08 mm^−1^).

### 2.6 Statistical Analysis

We conducted paired *t*‐tests to compare differences in tendon morphology and collagen organization between individuals with RC tendinopathy to their 1‐to‐1 matched asymptomatic controls, and in the RC tendinopathy group to compare shoulders (symptomatic to asymptomatic). The “involved” shoulder of the control was selected based on their matched patient's painful arm, and the “uninvolved” shoulder of the control was selected based on their matched patient's non‐painful arm. The following variables, in both groups, were analyzed for the involved and non‐involved side: tendon thickness and PSFR.

Normality assumptions were assessed via Shapiro–Wilk test, and Welch's *t*‐test was applied to account for unequal variances where necessary. Statistical significance was determined at *p* < 0.05. To assess relationships between tendon morphology and collagen organization, we examined Pearson correlation coefficients (r) for longitudinal tendon thickness vs. PSFR (involved and uninvolved sides). Statistical significance was determined at *p* < 0.05. To examine whether the relationship between PSFR and tendon thickness differed between the RC tendinopathy and Control groups, we conducted regression analyses with a cohort interaction. PSFR was the dependent variable, and tendon thickness and cohort were the predictors. An interaction term between tendon thickness and cohort was included to test whether the association between tendon thickness and PSFR varied by group. The models were specified as follows:

PSFR=β₀+β₁(TendonThickness)+β₂(Cohort)+β₃(TendonThickness×Cohort)+ϵ

where *β*₁ and *β*₂ represent the two predictors, *β*₃ represents the interaction term, and *ϵ* the error term. A statistically significant *β*₃ coefficient (*p* < 0.05) would indicate that the relationship between tendon thickness and PSFR is significantly different between the RC tendinopathy and Control groups.

## 3 Results

### 3.1 Group Comparisons

**FIGURE 3:**
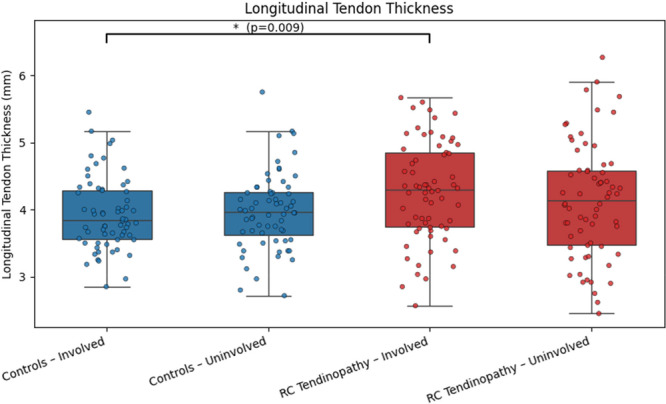
Boxplots comparing tendon thickness (mm) between RC tendinopathy participants and healthy matched controls. Boxes represent the interquartile range (IQR; 25th–75th percentile), with the median indicated by the horizontal line. Whiskers extend to the furthest data point within 1.5 × IQR from each quartile, and individual participant data points are shown as scattered dots.

**FIGURE 4:**
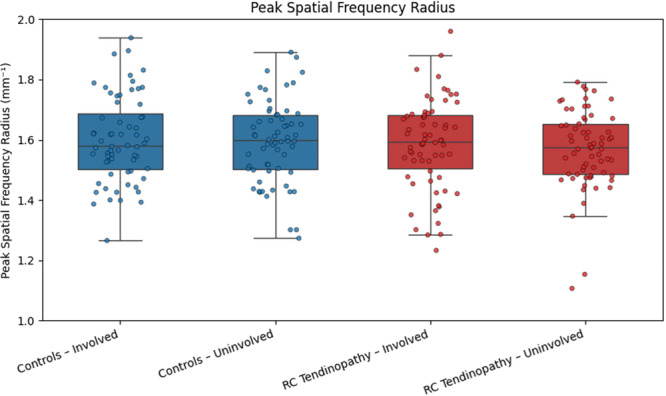
Boxplots comparing peak spatial frequency radius (PSFR, mm⁻¹) between RC tendinopathy participants and healthy matched controls. Boxes represent the interquartile range (IQR; 25th–75th percentile), with the median indicated by the horizontal line. Whiskers extend to the furthest data point within 1.5 × IQR from each quartile, and individual participant data points are shown as scattered dots.

Thickness and PSFR data demonstrated high variability in RC tendinopathy and controls (Figures 3 and 4). There was a statistically significant difference in longitudinal tendon thickness on the involved side, with individuals with RC tendinopathy exhibiting greater thickness compared to asymptomatic controls (Figure 3). The difference between groups for the uninvolved side was not significant (Figure 3). Collagen organization, as measured by PSFR, did not differ between groups (Figure 4). Thickness and PSFR data demonstrated high variability in RC tendinopathy and controls (Figures 3 and 4).

### 3.2 Bilateral Comparison

Participants with RC tendinopathy showed no significant differences between symptomatic and asymptomatic sides in tendon thickness or PSFR values. For longitudinal tendon thickness, the mean side‐to‐side difference was 0.15 mm (95% CI −0.04–0.29 mm; *p* = 0.12). For PSFR, the mean difference was 0.02 (95% CI −0.02–0.06; *p* = 0.4).

Similarly, controls demonstrated no significant bilateral differences. The mean side‐to‐side difference in longitudinal tendon thickness was 0.02 mm (95% CI −0.03–0.07 mm; *p* = 0.45), and for PSFR the mean difference was 0.01 (95% CI −0.04–0.05; *p* = 0.76).

### 3.3 Correlational Analysis

**FIGURE 5:**
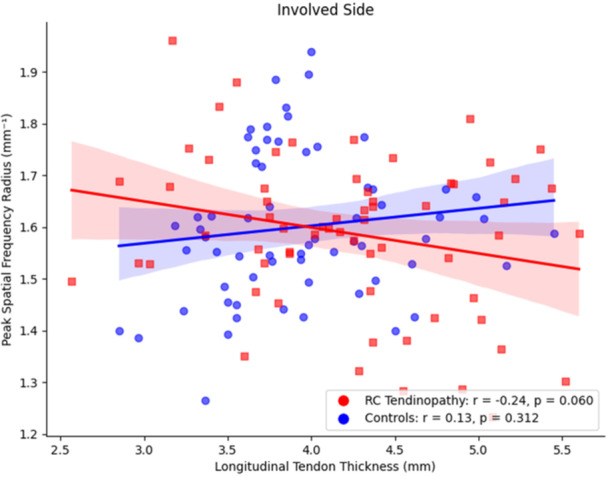
Relationship between involved side tendon thickness in the long axis and peak spatial frequency radius in each group.

**FIGURE 6:**
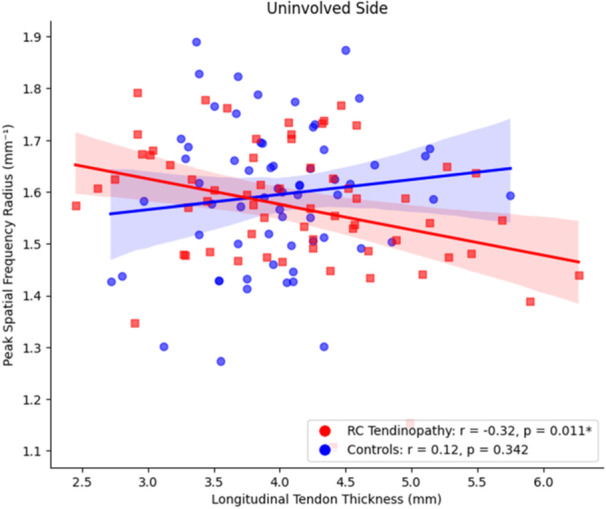
Relationship between uninvolved side tendon thickness in the long axis and peak spatial frequency radius in each group.

Within the RC tendinopathy group, a negative correlation was found between tendon thickness and PSFR on the involved side (*r* = −0.24, *p* = 0.060) (Figure 5) and non‐involved side (*r* = −0.32, *p* = 0.01) (Figure 6), indicating that greater tendon thickness may be associated with lower collagen organization in patients with RC tendinopathy, both on the symptomatic and asymptomatic shoulders.

Within the control group no significant relationships were found between tendon thickness and PSFR for the involved (*r* = 0.13, *p* = 0.3) and non‐involved sides (*r* = 0.12, *p* = 0.3).

### 3.4 Regression Analysis

The interaction between tendon thickness, PSFR and group was significant on both the involved (*β* = −0.054, *p* = 0.050) and the uninvolved side (*β* = −0.056, *p* = 0.027), indicating that the association between tendon thickness and PSFR varied by group (cohort). In both cases, the results indicate that the relationship between tendon thickness and PSFR varied between the RC tendinopathy and Control groups. Full model results are included in Supporting Material 1.

## 4 Discussion

This study examined the differences in tendon thickness and collagen organization between individuals with unilateral symptomatic RC tendinopathy and asymptomatic controls, as well as bilateral differences in patients with unilateral RC tendinopathy, and examined the correlation between the two tendon structural metrics in those with tendinopathy. Participants with RC tendinopathy demonstrated thicker tendons on the involved side compared to controls. Notably, the relationship between tendon thickness and micromorphology differed between groups: in healthy individuals, greater tendon thickness was not associated with alterations in micromorphology, suggesting preserved structural integrity. In contrast, in individuals with RC tendinopathy, greater tendon thickness was accompanied by greater collagen disorganization, as evidenced by lower PSFR in both the symptomatic and asymptomatic shoulders. To our knowledge, this is the first study to examine both structural levels concurrently, providing a robust structural profile of tendon health.

Bilateral comparisons of tendon structural metrics between shoulders in those with RC tendinopathy revealed no differences. We hypothesized a thicker and more disorganized tendon on the painful side in those with tendinopathy. This lack of differences may reflect tendon adaptation in the asymptomatic shoulder that has not reached a threshold of pain and maladaptation. The dominant shoulder is inferred to be used more frequently in activities and thus expected to be more commonly the painful shoulder in those with tendinopathy. However, 34% of the participants with RC tendinopathy report pain on their non‐dominant side.

Interestingly, the negative correlation between tendon thickness and PSFR observed in both the symptomatic and asymptomatic shoulders of the tendinopathy group was not present in healthy controls. This divergence may reflect underlying differences in the biological mechanisms driving tendon thickening. In individuals with RC tendinopathy, greater tendon thickness may result from degenerative changes, including inflammation, increased water content, and collagen disorganization—factors that collectively, or by themselves individually, contribute to lower PSFR values. In contrast, healthy controls may exhibit greater tendon thickness because of adaptive hypertrophy from loading with activities. This physiological thickening likely occurs without the accompanying structural degradation, allowing the collagen matrix to remain organized and PSFR values to remain stable. These findings suggest that tendon hypertrophy in the presence of pathology may reflect maladaptive remodeling, while in healthy individuals, it represents a beneficial structural adaptation.

We found 0.4 mm greater tendon thickness in those with RC tendinopathy as compared to healthy matched controls. The relevance of this thickness can be considered in relationship to prior studies. In a longitudinal study of 8‐week resistance exercise [29] for every 1 mm of tendon thickness reduction there was a 14.4‐point increase (improvement) in shoulder disability (Penn). Moreover, the MDC for our method of measuring tendon thickness is 0.3 mm, thus 0.4 mm difference is greater than measurement error.

Although differences in tendon thickness have been shown to differentiate individuals with tendinopathy from healthy controls, our findings may reflect a more complex relationship than anticipated. According to the continuum model of tendinopathy, pathology can be classified into at least two subgroups: reactive tendinopathy, typically marked by tendon thickening and hypoechoic regions, and degenerative tendinopathy, often associated with tendon thinning and structural breakdown [30, 31, 32, 33]. Notably, a recent study by Dube et al. [34] demonstrated that supraspinatus tendon thickness responds differently to exercise depending on the specific tendinopathy subtype. In our study, we did not differentiate participants based on these subgroups, which may have diluted the expected correlations between tendon thickness and PSFR. In addition, symptom duration ranged widely (1 month to 10 years), which may have led to a patient cohort mix of reactive and degenerative tendinopathy. It is possible that opposing structural adaptations within reactive vs. degenerative tendinopathy—thickening vs. thinning—resulted in a net attenuation of the observed relationship. Future research should consider subgroup classification to better capture these contrasting structural patterns and their relationship to tendon micromorphology.

Our results were like the previous findings of Pozzi et al. [10]. When comparing healthy controls with those with RC tendinopathy, there was no significant difference in mean PSFR values. A possible explanation for this unexpected finding in both studies is that some patients may not have detectable collagen organizational damage, and only have inflammation causing tendon thickening. Our study also supports previous studies [4, 5, 6, 7, 14], showing that individuals with RC tendinopathy exhibit a modestly thicker tendons compared to healthy controls. However, a unique finding in this study was in individuals with RC tendinopathy there was an inverse relationship between tendon thickness and PSFR, indicating with increased tendon thickness the collagen organization worsens.

There are limitations to this study. The participants had relatively low levels of pain (~3/10 pain rating on average), thus potentially less likely tendon structural damage. On the other hand, our participants did have at least 3 months of shoulder pain. Notably, PSFR may be less effective as a standalone metric in the supraspinatus tendon compared to its use in other tendons, such as the Achilles and patellar tendons. This discrepancy is likely attributed to key anatomical and functional differences—namely, the supraspinatus's exposure to increased compressive and shear force, more heterogeneous collagen organization higher water content, and proximity to synovial fluid—all of which may influence how micromorphological changes manifest in this tendon. Continued investigation into the supraspinatus and its morphological adaptations should include multiple metrics to accurately interpret change.

Overall, our findings enhance the understanding of how tendon thickness and collagen organization interact to distinguish pathological from healthy tissue. Moreover, we provide a unique finding; tendon thickening is related to collagen disorganization in the supraspinatus tendon of patients with RC tendinopathy. By independently and jointly characterizing tendon macro‐morphology and micro‐morphology, we provide valuable insight into the complex structural changes that occur in the tendon.

## Author Contributions

All authors meet the criteria for authorship based on substantial contributions to the work. Lori A. Michener and Oscar Vila‐Dieguez conceptualized and designed the study. M. Chris Zipser, Oscar Vila‐Dieguez, Kira Chow, and Lori A. Michener were responsible for data acquisition and analysis. M. Chris Zipser drafted the initial manuscript. All authors critically revised the manuscript for important intellectual content. All authors reviewed and approved the final submitted version of the manuscript.

## Supporting information

Supporting Material 1.
