# Supplementary material for: Supraspinatus Tendon Thickness Is Associated With Collagen Disorganization in Symptomatic and Asymptomatic Shoulders of Individuals With Unilateral Rotator Cuff Tendinopathy
Source: J Orthop Res. 2026 Apr 3;44(4):e70200. doi: 10.1002/jor.70200 (PMC13047412; doi:10.1002/jor.70200)
Supplement: Supplementary file 1 — Supporting Material 1. [file JOR-44-0-s001.docx]

**Involved Side Model**

**Model:**PSFR = β₀ + β₁(TT_z) + β₂(Cohort) + β₃(TT_z × Cohort) + ε

- Coefficients:

| Term | β | SE | p-value |
| --- | --- | --- | --- |
| Intercept (β₀) | 1.6051 | 0.019 | <0.001 |
| Thickness (β₁) | 0.0217 | 0.0218 | 0.321 |
| Cohort (β₂) | -0.0098 | 0.0262 | 0.710 |
| Interaction (β₃) | -0.0541 | 0.0274 | 0.050 |

Residual SD: 0.1446

R²: 0.039

n: 128

**Uninvolved Side Model**

**Model:**PSFR = β₀ + β₁(TT_z) + β₂(Cohort) + β₃(TT_z × Cohort) + ε

- Coefficients:

| Term | β | SE | p-value |
| --- | --- | --- | --- |
| Intercept (β₀) | 1.5958 | 0.0164 | <0.001 |
| Thickness (β₁) | 0.0206 | 0.0207 | 0.320 |
| Cohort (β₂) | -0.0216 | 0.0231 | 0.353 |
| Interaction (β₃) | -0.0558 | 0.0250 | 0.027 |

Residual SD: 0.1305

R²: 0.062

n: 128
